# Editorial: Breaking barriers to diversify the physician workforce

**DOI:** 10.3389/fpubh.2023.1190382

**Published:** 2023-05-16

**Authors:** Sylk Sotto-Santiago, Inginia Genao, Meshell Johnson

**Affiliations:** ^1^School of Medicine, Indiana University Bloomington, Indianapolis, IN, United States; ^2^College of Medicine, The Pennsylvania State University, Hershey, PA, United States; ^3^San Francisco Veterans Affairs Health Care System and School of Medicine, University of California San Francisco, San Francisco, CA, United States

**Keywords:** diversity and inclusion, equity, physician workforce diversity, Black and Hispanic/Latino, racial climate

In recent years, we have seen increased attention to diversity, equity, and inclusion in academic medicine in the U.S. In fact, higher education institutions have increasingly implemented formal diversity initiatives over the past 50 years to support students from historically and racially underrepresented groups (1). In the 1960s, student demands for institutional accountability set the path forward for multicultural/diversity offices along with leaders charged with providing student support. In the 1980s and 1990s, institutions were motivated by the foundations of multicultural education inspiring curricular change to counter educational and social injustice (1, 2). Closer attention also resulted in an examination of the experiences of students of color in higher education, summarized by hostile and unwelcoming environments (3). The result was a rise in diversity leaders hired to create and lead the institutional diversity plan and strategic direction (1, 4).

Unfortunately, academic medicine has a long history of racism, discrimination, and ethical atrocities that were committed in the name of medicine. The Flexner Report (1910), a commentary on medical education that promoted segregation and severely limited the ability of Black physicians to train, had a lasting impact in medical education, considerably damaging historically Black medical schools and serving as a prime example of systemic racism (5). The changing demographics of the U.S. and the vast health inequities and disparities among racial groups continued to be highly visible. The number of historically and underrepresented providers across all of medicine remained dismal. In 2003, the Sullivan Commission on Diversity in the Healthcare Workforce provided recommendations to address the lack of diversity in the health professions and ways to promote systemic change (6). The *Missing Persons: Minorities in the Health Professions* report emphasized that diversifying the health professions had focused on recruitment and retention of students of color, but that diversity must be considered more broadly, inclusive of faculty and staff, and observed in the areas of education, clinical and research activities, and public missions of institutions (7). The report also characterized the importance of medical school leadership and faculty in ensuring that an institution's policies align with its mission, and that they reform medical education by overseeing the recruitment, retention, and promotion of students and faculty of color (6). Medical education reform has included the adoption of cultural competency as a means to improve the quality of care that clinicians deliver to minoritized patient populations. In the late 1990s, cultural humility also offered a way to encourage self-reflection, lifelong learning, and a check of power imbalances to develop meaningful communication and mutually respectful relationships with patients. More recently, medical education reform is now focusing on antiracism, health equity, and other concepts that highlight structural and social determinants or drivers of health (7). Sadly, the underrepresentation of students and faculty of color has not changed significantly since the institution of the 2009 Liaison Committee of Medical Education diversity accreditation guidelines (8–11). Yet, scholars have continued to report a misalignment between the values professed by medical institutions and population health outcomes. In addition, there is a perceived disconnect between what drives institutions and the mission of social responsibility in academic medicine (12, 13).

Progress is relative. The AAMC's 2015 report: *Altering the Course: Black Males in Medicine*, noted the number of Black men enrolled in medical school actually decreased between 1978 and 2014. In 1978, 1,410 Black males applied to medical school, and in 2014, just 1,337 applied (14). As this data continue to be monitored, the percentage of Black men in medical schools has increased only slightly (15). In 2019, only 5.3% of graduates from U.S. medical schools identified as Hispanic/Latinx or of Spanish origin, but the number of Black, Hispanic, and women applicants and enrollees increased at U.S. medical schools in 2022 (16, 17). We continue to work toward elevating the voices of historically and underrepresented groups in higher education and frankly, facing what some may consider an attack to these efforts in recent years, such as the pending cases before the U. S. Supreme Court: Students for Fair Admissions v. Harvard and Students for Fair Admissions v. University of North Carolina, seeking to prohibit the limited consideration of an applicant's racial or ethnic background in the higher education admissions process (18, 19).

The importance of this Research Topic: *Breaking Barriers to Diversify the Physician Workforce* cannot be understated. It is known that the physician and healthcare workforce does not reflect the patient population we serve. Efforts exist to diversify the physician workforce, especially in relationship to racially and historically underrepresented groups, along with socio-economic diversity. However, in addition to these efforts and scholarship, there needs to be better guidance on strategies and best practices for retaining physicians of color, as well as caring for racially minoritized populations.

We sought articles that included historical perspectives on forms of racism, discrimination, and the systems that support them still today. We are proud to present eigh articles and 29 authors who are doing the critical work needed. With their unique contributions they offered a look at key practices for search committees, DEI committee structure within graduate medical education, and admissions. Scholars were able to present a professional development program that incorporated best practices to promote diversity at each stage of the search process (Jacobs et al.). Moreover, scholars provided insight into the establishment of DEI committees that have implications for support of institutional recruitment efforts and inclusive environments (Kara et al.). In addition, Joy demonstrated the value of intersectionality and how harnessing the power of holistic reviews can mitigate bias throughout the admissions process. Walker and Williams continue to highlight the importance of mentoring as a meaningful contribution toward the diversification of the physician workforce through equity-focused mentoring models.

Additional articles pay close attention to underrepresented groups in medicine. Corsino et al. looked at Hispanic, Latinx, and/or Spanish origin experiences and acknowledged the required multifaceted approach to their success while highlighting the ongoing challenges that limit students from pursuing and completing medical school. Salinas et al. challenged us to look at first generation/low income experience of those navigating academic medicine. We need to center the work that we are doing advancing DEI on the lived experiences of the populations that we are intending to serve. Sharp et al. explicitly state that the lack of intention behind addressing the “needs of Black women can be viewed as complicity in the oppressive structures that serve to subjugate them”.

Lastly, we agree with Esparza et al. The authors highlight a key aspect of this special issue. Medicine has a long history of its own. Without intentional looks at the medical education system, we risk perpetuating exclusion, racism, discrimination, and harm, revealing environments that are “near impossible to enter and incredibly traumatic for those with multiple marginalized identities. Until we approach DEI in academic medicine with the rigor, intentionality, and investment needed to evaluate and influence an exceedingly complex and adaptable ecosystem of oppression, meaningful change will continue to elude us”.

Despite the challenges we face, through this special issue, we have provided literature, practices, and perspectives that impact the diversification of the physician workforce. As we continue to aim for diversity, inclusion, and belonging, there are innovative ways to move forward in each of these areas with research and practice in our fields.

## Author contributions

IG and MJ contributed to the direction and writing and edits of all versions of the manuscript. All authors read and approved the final manuscript and contributed to this editorial.
